# Supplementary material for: Surface properties of Ga–Cu based liquid-metal alloys: impact of Cu dilution, topography, and alloy liquefaction
Source: RSC Appl Interfaces. 2026 Jan 6;3(2):413–9. doi: 10.1039/d5lf00335k (PMC12818026; doi:10.1039/d5lf00335k)
Supplement: LF-003-D5LF00335K-s001 [file LF-003-D5LF00335K-s001.pdf]

Supplementary Information (SI) for RSC Applied Interfaces.
This journal is © The Royal Society of Chemistry 2026

Supporting Information

Surface Properties of Ga-Cu Based Liquid-Metal Alloys: Impact of Cu Dilution, Topography, and Alloy Liquefaction

Tzung-En Hsieh[a†], Michael S. Moritz[b†], Andreas Mölkner[c†], Christoph Wichmann[b, d], Johannes Frisch[a, e], Julien Steffen[c], Caiden J. Parker[f], Vaishnavi Krishnamurthi[f], Torben Daeneke[f], Hans-Peter Steinrück[b], Andreas Görling[c, g], Christian Papp[d], Marcus Bär[a, e, h, i]

[a]*Department Interface Design, Helmholtz-Zentrum Berlin für Materialien und Energie GmbH (HZB), 12489 Berlin, Germany*

[b]*Friedrich-Alexander-Universität Erlangen-Nürnberg (FAU), Lehrstuhl für Physikalische Chemie, 91058 Erlangen, Germany*

[c]*Friedrich-Alexander-Universität Erlangen-Nürnberg (FAU), Lehrstuhl für Theoretische Chemie, 91058 Erlangen, Germany.*

[d]*Freie Universität Berlin, Angewandte Physikalische Chemie, 14195 Berlin, Germany*

[e]*Energy Materials In-situ Laboratory Berlin (EMIL), HZB, 12489 Berlin, Germany*

[f]*School of Engineering, RMIT University, 3000 Melbourne, Australia*

[g]*Erlangen National High Performance Computing Center (NHR@FAU), D-91058 Erlangen, Germany*

[h]*Department of Chemistry and Pharmacy, Friedrich-Alexander-Universität Erlangen-Nürnberg (FAU), 91058 Erlangen, Germany*

[i]*Department X-ray Spectroscopy at Interfaces of Thin Films, Helmholtz-Institute Erlangen-Nürnberg for Renewable Energy (HI ERN), 12489 Berlin, Germany*

Table of Contents:

Experimental Section S1
Figure S1. Illustration of microscopic and macroscopic model systems S10
Figure S2. Survey of microscopic Ga-Cu model systems S11
Figure S3. Fits of the Ga 3d XPS spectra of microscopic Ga-Cu model systems S12
Figure S4. Fits of the Cu $2p_{3/2}$ XPS spectra of microscopic Ga-Cu model systems S13
Figure S5. Different UP spectra of 5 at% and 1 at% Cu containing Ga-Cu model systems S14
Figure S6. Schematic illustration of ML-FF calculated Ga-Cu unit cells S15
Figure S7. Schematic illustration of ML-FF calculated unit cell of Ga_2Cu IMC S16
Figure S8. DSC measurement of Ga-Cu sample with 1wt% Cu S17
Figure S9. In-situ annealing TEM image of Ga-Cu nano-droplets with 5 wt% Cu S18
Figure S10. Calculated radial distribution function plots of Ga-Cu unit cells S19
Figure S11. Calculated integrated radial distribution function plots of Ga-Cu unit cells S20
Figure S12. Calculated integrated radial distribution function plots of Ga_2Cu IMC S21
Figure S13. In-situ annealing XP Ga core level spectra of microscopic Ga-Cu model system S22
Figure S14. SEM image of microscopic Ga-Cu model system after 400 °C annealing S23
Figure S15. Temperature dependent XP survey spectra of macroscopic Ga-Cu model system S24
Figure S16 Temperature dependent XP Ga 3d and Cu $2p_{3/2}$ spectra of macroscopic Ga-Cu model systems S25
Figure S17 XP Ag $3d_{5/2}$ and Au 4f spectra of microscopic Ga-Ag and Ga-Au model systems S27
Figure S18 XP survey spectra of microscopic Ga-Ag model system S27
Figure S19. XP survey spectra of microscopic Ga-Au model system S28
Figure S20. Ga $2p_{3/2}$ spectra of pure Ga and nano-scale Ga-Ag and Ga-Au model system S28
Figure S21. Fits of the Ag $3d_{5/2}$ XPS spectra of pure Ag and nano-scale Ga-Ag model systems S29
Figure S22. Fits of the Au 4f XPS spectra of pure Au and nano-scale Ga-Ag model systems S30
Figure S23. Summary of temperature dependent XP spectra and Ag/Au concentrations of macroscopic Ga-Ag and Ga-Au model systems S31
Figure S24. Temperature dependent XP Ga 3d and Ag $3d_{5/2}$ spectra of macroscopic Ga-Ag model systems S34
Figure S25. Temperature dependent XP Ga 3d and Au 4f spectra of macroscopic Ga-Au model systems S35
Figure S26. Temperature dependent XP survey spectra of macroscopic Ga-Ag model systems S36
Figure S27. Temperature dependent XP survey spectra of macroscopic Ga-Au model systems S37
Table 1. TEM derived Ga_2Cu IMC dissolution for a Ga-Cu nanodroplet with 10 wt% Cu S38
References S39

Experimental Section

Preparation of macroscopic model systems

The macroscopic model systems for X-ray photoelectron spectroscopy (XPS) measurements were prepared by weighing in the respective amounts of Ga (Sigma-Aldrich, 99.9995%) and adding the transition metal (TM: Cu - MaTeck, 99.995%; Ag - MaTeck, 99.9%; Au - MaTeck, 99.99%) in low quantities aiming for a nominal TM concentration of 1 at.%. The samples were supported on a W boat crucible (Kurt J. Lesker Company) and weighed roughly 1 g. Ta wires and type K Ni/NiCr thermocouples were spot welded to the bottom of the W boat for resistive heating and temperature reading. The accuracy of the temperature reading was determined to be ±2° C using the melting temperature of Ga.[1] The samples were cleaned by heating to 427 °C and Ar^+ sputtering (1 keV, 10^{-5} mbar). The surface was checked by XPS (O 1s, C 1s, and Ga 2p regions) to check for surface contaminants.

For differential scanning calorimetry (DSC) measurements macroscopic model system samples were prepared based on Ga metal with a purity of 99.9% purchased from Indium Corporation, while Cu powder with a purity of 99.9% was obtained from Sigma-Aldrich. To prepare a 1wt% Ga-Cu bulk sample, the Cu powder was mixed with Ga metal at a temperature of 400 °C using a mortar and pestle. This process was performed in a nitrogen glovebox to minimize oxidation during mixing. Once the sample was prepared, the 1wt% Ga-Cu was allowed to freeze at -80 °C and was subsequently stored in a fridge at 5 °C in solid form until performing DSC analysis.

Preparation of microscopic model systems

The microscopic model systems for the XPS studies were prepared by a co-deposition process using physical-vapor-deposition (PVD). Ga powder (Sigma-Aldrich, 99.99 %) as well as Cu powder (Thermo Fischer, 99.999 %), Ag rods (Sigma-Aldrich, 2mm diameter, 99.95%), and

cut Au foil (0.2 mm thick, Sigma-Aldrich, 99.99%), respectively, were employed as targets for the Ga-TM sample depositions. The PVD of the Ga-Cu, Ga-Ag, and Ga-Au model system samples have been conducted with a SPECS EBE-4 e-beam evaporator. All samples were prepared in ultra-high vacuum (UHV) at a base pressure < 1×10^{-8} mbar by co-evaporation of Ga/Cu, Ga/Ag and Ga/Au on a natively oxidized silicon wafer (Boron doped, 2-4 Ω·cm resistance, Czochralsky) referred to as SiO_x/Si. Substrates were annealed at 500 °C for 30 minutes to clean the support surface and desorb water before deposition. For all Ga-TM samples a nominal film thickness of 30 nm is aimed for, the deposition rate is acquired by TMC13 quartz micro balance from Prevac.

For transmission electron microscopy (TEM) two Ga-Cu bulk samples were prepared in the same way as for the DSC measurements, albeit with higher Cu concentration of ≈ 5 and 10 wt%. The nanodroplet synthesis then began by heating 15.3 g anhydrous NaOAc (99%, sourced from Sigma Aldrich) to its melting point within a vial contained by an aluminum heating block. The temperature is maintained at 400 °C on a hotplate equipped with a thermocouple. Once all of the NaOAc is liquid, 1 g of Cu-Ga bulk sample is added and stirred at 250 rpm using a magnetic stirrer. Following this, a SCIENTZ-IID probe sonicator equipped with a 6 mm titanium tip is lowered into the molten NaOAc and allowed to thermalize. Sonication then ran for 30 minutes at 300 W power accompanied with a 3-second on, 3-second off sonication pattern with stirring maintained at 250 rpm concurrently. Upon cooling, the NaOAc was allowed to solidify. Once warm to the touch, the molten salt containing Ga-Cu nanodroplets is washed with approximately 500 ml of deionized water. The resulting droplets are then isolated *via* vacuum filtration through PETE membranes with a pore size of 400 nm and a diameter of 47 mm from Sterlitech. After three washing cycles the sample was redispersed in a 90 vol% ethanol-water solution (with ethanol of purity 99.5% procured from Thermo Fisher Scientific). This solution was then drop cast onto a Protochips e-chip with a

carbon membrane for in-situ TEM analysis while increasing temperature. The 5wt% Ga-Cu sample was used for imaging, while the 10wt% Ga-Cu sample was employed to determine the solubility of Cu in Ga at different temperatures.

Preparation of pure transition metal references

Cu and Ag reference films on SiO_x/Si supports are prepared by PVD using the same set-up as used for the microscopic model system preparation. Ar^+ sputter-cleaned (1×10^{-5} mbar Ar, 800 eV, 10 mA emission current) Au foil is utilized as Au reference.

XPS of the macroscopic model systems

XPS of the macroscopic Ga-Cu, Ga-Ag, and Ga-Au model system samples was measured with a dedicated instrument described in detail in literature.[2] The instrument is equipped with a modified OMICRON EA 125 hemispherical electron kinetic energy analyzer and a non-monochromatized SPECS XR-50 NAP Al/Mg dual X-ray anode; for the measurements, Al K_α (1486.58 eV) was used. The X-ray tube is separated from the measurement chamber by a 0.2 μm thick Si_3N_4 window and the electron optics and analyzer are separated from the measurement chamber by differential pumping stages. The binding energy of core level peaks was calibrated by setting the Fermi-edge to 0.0 eV.

XPS/UPS of the microscopic model systems

X-ray (XPS) and ultraviolet (UPS) photoelectron spectroscopy measurements of the microscopic Ga-Cu, Ga-Ag, and Ga-Au model systems were conducted using non-monochromatized Mg K_α (1253.56 eV) irradiation from a SPECS XR 50 X-ray source and He II (40.8 eV) irradiation from a Prevac UVS 40A2 gas discharge lamp, respectively. The photoelectrons were detected by a ScientaOmicron Argus CU electron analyzer. The pass energy for the core level detail spectra measurements was set to 20 eV, resulting in a total experimental energy resolution of approximately 0.7 eV for Mg K_α-XPS. For the He II-UPS

measurements, a pass energy of 5 eV was used, resulting in a total experimental resolution of approximately 0.2 eV. The binding energy (BE) of the XPS and UPS measurements was calibrated by the Fermi-edge (E_F) of a sputter-cleaned Au foil.

TEM and SEM measurements of microscopic model systems

The scanning electron microscopy (SEM) measurements on microscopic Ga-Cu model systems on SiO_x/Si supports are conducted by Zeiss Merlin SEM with a Schottky field emission source and in-lens detector.

The TEM measurements on Ga-Cu nanodroplets were conducted by a specialized holder (Fusion holder, designed by Protochips) allowing for in-situ annealing experiments. This enabled a close monitoring of the dissolution of the Cu inside the Ga metal. TEM images of the Ga-Cu nanodroplets were acquired by the JEOL F200 instrument at an operating voltage of 200 kV. Gatan Digital Micrograph (version 3.43.3213.0) was utilized for image capturing from the Rio16 4k charge-coupled device camera (model 1816) followed by analysis. Elemental composition was derived by energy-dispersive X-ray spectroscopy (EDS) using an Oxford X-Max^n 80T X-ray spectrometer ran by Aztec software. EDS (maps) have been collected at 200 kV with an energy range of 40 keV to capture all relevant peaks, with an acquisition time of 30 minutes per droplet, process time of 6, and a resolution/channels of 2048.

DSC analysis of microscopic model systems

DSC measurements of Ga-Cu bulk samples with 1wt% Cu were conducted using a Pyris 1 calorimeter (Perkin Elmer). The experiments were carried out in ceramic pans under a nitrogen atmosphere with a sample amount of ~ 6 mg. A heating rate of 10 °C/min was applied, with temperatures ranging from 20 °C to 400 °C. The baseline subtraction was performed using the

DSC Pyris Software. The melting/crystallization points were defined as the onset of the peaks observed after DSC scan.

XPS/UPS data evaluation

The XPS data collected for the macroscopic model systems were evaluated using CasaXPS Version 2.3.18PR1.0. For asymmetric metallic signals, a Lorentzian Finite (LF) lineshape, as implemented in CasaXPS, was used.[3] Symmetric and oxidic peaks were fitted with a Gaussian-Lorentzian product function (GL(70)). Line shapes and spin-orbit splitting were first optimized with pure metal reference samples and then adapted. The peak areas were normalized for quantification by dividing by the corresponding sensitivity factors. These factors are calculated by the product of the core-level specific photoionization cross-section σ, inelastic mean free path λ_{IMFP}, and the inverse kinetic energy of the photoelectron as an instrument-specific transmission function.

$$\frac{I}{SF} = \frac{I}{\sigma \cdot \lambda_{IMFP} \cdot E_{kin}^{-0.33}}$$

The XPS/UPS data of the microscopic model systems were fitted and quantified by Winspec (LISE, Université de Paix, Namur). The XPS peaks of metallic components (Ag, Au, Cu, Ga) are fitted by asymmetric (Doniach-Sunjic) profiles; those of oxides (GaO_x, AgO_x) are fitted by Voigt profiles. The fitting includes a Shirley background for quantitative analysis. The peak width and position and intensity ratio of spin-orbit split doublet peaks are coupled in all fitting routines and the spectrum is fitted sequentially with fixed peak shape and width. The derived area of the core level peaks studied is corrected for the energy-dependent transmission function of the used analyzer using the following equation:

$$I = I_0 * (0.61 + 0.00021 * (E_x - E_b)$$

I and I_0 denote the calibrated peak area and original peak area, respectively. E_x denotes the excitation energy (Mg K_α = 1253.56 eV), E_b denotes to the binding energy of the core level peak. For quantifications, the respective peak areas were additionally corrected by the respective sensitivity factors. These factors are the product of the photoionization cross section σ,[4-6] and the inelastic mean free path λ_{IMFP} of the photoelectrons provided by Sessa v2.2.[7-10] The quantitative analyses of certain core level peaks or elements in this study are then processed by the following equation:

$$\frac{A}{B} = \left(\frac{IA}{\sigma A} \div \frac{IB}{\sigma B} \div \frac{\lambda A}{\lambda B}\right)$$

I_A and I_B denote the peak area of core levels A and B. σ_A and σ_B are the respective photoionization cross sections of core levels A and B. λ_A and λ_B denote to inelastic mean free path of photoelectrons of core level A and B, respectively.

Machine Learning Force Field (ML-FF) Calculations

All calculations and simulations were performed with the *Vienna Ab-initio Simulation Package* (VASP) version 6.4.2, which describes the valence electrons with plane waves and represents the atomic cores with the projector augmented wave (PAW) method.[11-12] For the generation of the machine-learned force fields (ML-FF) and all further DFT calculations the following settings were used.[13] The functional of Perdew, Burke and Ernzerhof (PBE) was used to approximate the exchange-correlation energy.[14] The energy convergence criterion was set to 10^{-7} eV and the maximum gradient criterion of the ionic relaxation was set on 10^{-2} eV/Å. The energy cut-off of the plane wave basis set was set to 310 eV for the ML-FF training and the core level shift calculations but was increased to 415 eV for the density of states (DOS) calculations. Furthermore, Methfessel-Paxton smearing of the first order for electronic states with the smearing width set to 0.2 eV and the DFT-D3 dispersion correction of Grimme was

used.[15-16] For all calculations a 2x2x1 k-point mesh was used for the sampling of the reciprocal space. This was increased to 4x4x1 k-point mesh for the DOS calculations.

Two ML-FFs were trained for this study. One for simulations with 1% Cu and one for 36% Cu loading. The first was trained with a unit cell consisting of 200 atoms placed on a simple cubic grid with 180 Ga and 20 Cu (10%) atoms. The training simulation consisted of 250.000 steps of 2 fs length at a constant temperature of 1000 K. The number of local reference configurations was set to 6500 with the option for configurations to be replaced during the simulation. The number of stored reference structures increased automatically during the simulation. The Andersen thermostat was used due to beneficial effects on the stability of the training simulation. The ML-FF radial and angular descriptor cutoff were set to 8 Å.[17] Afterwards, the force-field was refitted to the fast version without uncertainty prediction.

The second force field was trained with a unit cell consisting of a Cu 111 surface slab with 4 layers (120 Atoms in total) and 150 Ga Atoms placed on top in a simple cubic grid. The number of steps and the temperature were increased to 500.000 steps and 1500 K, respectively. The thermostat was switched to the Nose-Hoover thermostat.[18-19] The other settings were kept from the first training simulation. After the 500.000 steps were reached the force field was refitted to the fast version with the angular descriptor cutoff reduced to 5 Å.

The refitted force fields were used to simulate five liquid cells each. The cells of the first force field contained 180 atoms in total with two of them being Cu (1.1%) and 178 Ga. All cells had dimensions 12.69 × 12.69 × 36.15 Å. For the second force field the Cu content was increased to 65 Cu atoms. Each of those cells was simulated for 8.000.000 steps of 2 fs length at 300 and 800 K with the Nose-Hoover thermostat. The high Cu content cells were annealed for 1.000.000 at the respective temperature before.

The unit cell of the Ga_2Cu IMC was multiplied (4x4x2) and geometry optimized. The final structure of the optimization was used to calculate the Cu 2p core level shifts for each of the 32 Cu atoms inside the cell.

To study the Ga_2Cu IMC, NpT on-the-fly machine learning simulations were performed with the multipled (4×4×2) cell (Fig. S7, S12) at 300 and 800K for 100000 steps of 2fs length. For all NpT simulations the angles of the cells were kept fixed. The final structures and the Cu $2p_{3/2}$ core level shift (CLS) of the final structure at 800K can be seen in Fig. S12. The CLS have been calculated for each Cu atom of the final structure. Follow up simulations at 800K for additional 200000 steps revealed complete melting of the IMC. The melted structure is depicted in Fig. S12. Furthermore, a NVT on-the-fly machine learning simulation of a multiplied (6x6x3) cell was performed for 100000 steps of 2fs at 800K. The resulting force field was refitted and used for NVT and NpT simulations of a multiplied (8x8x4) cell at 300 and 800K to calculated RDFs and IRDFs if of the IMC. The simulations consisted of 1000000 steps of 2fs. At 300K the IMC stayed stable for both simulation types. At 800K the IMC melted in the case of the NpT simulation and stayed stable in the NVT case. The resulting NVT IRDFs is plotted in Fig. S12.

From the combined trajectories of each temperature the elemental density distribution along the z-axis of the cell was calculated. For the calculation of Cu 2p core level shifts snapshots were extracted from the trajectory. The snapshots were picked by dividing the simulation cell in 100 slices along the z-axis and by searching in the trajectory for structures with at least one Cu atom in the slice. This leads to a set of structures that contain each possible z-position of the Cu atom. To increase the number of snapshots, each trajectory was cut into five parts and each of those parts was analyzed separately in the described way.[20]

For each snapshot the Cu 2p core level shifts were calculated with the Janak-Slater transition state method.[21-23] The resulting eigenenergies of the respective orbitals were related to the calculated Fermi level of the respective calculation. Since absolute energies are of little use due to the PAW method for the atomic core region, the shifts are calculated in respect to a calculated reference $2p_{3/2}$ energy of a solid Cu crystal. The resulting chemical shifts were added to the experimental binging energy of Cu $2p_{3/2}$ of 932.6 eV and each was Gaussian broadened with a width of 0.03 eV. The results were evaluated in a histogram.

For the calculation of the DOS of pure Cu, a Cu (111) surface was used. The structures of the 1% and 36% Cu containing Ga-Cu samples were picked from respective simulation at 300° K. For each concentration 10 structures were picked. Each of these structures had one atom near the surface and one (or more) atoms in the bulk of the cell in order to investigate the difference in DOS for the surface and the bulk. The resulting partial density of states was averaged.

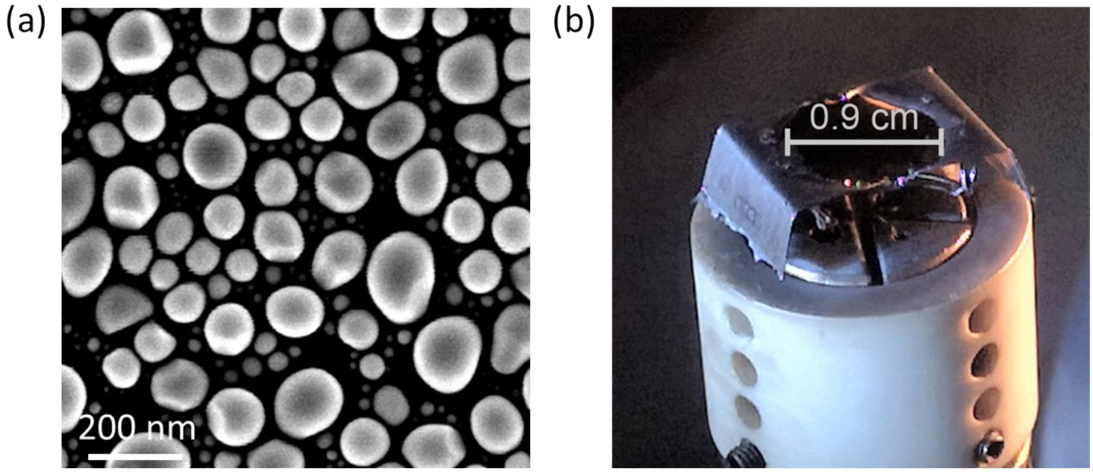

Figure S1. Exemplary illustration of the microscopic and macroscopic model system samples studied by XPS: (a) SEM image of a 5 at% Cu containing SiO_x/Si supported Ga-Cu microscopic model system sample and (b) the photograph of a 1 at% Cu containing Ga-Cu macroscopic model system sample.

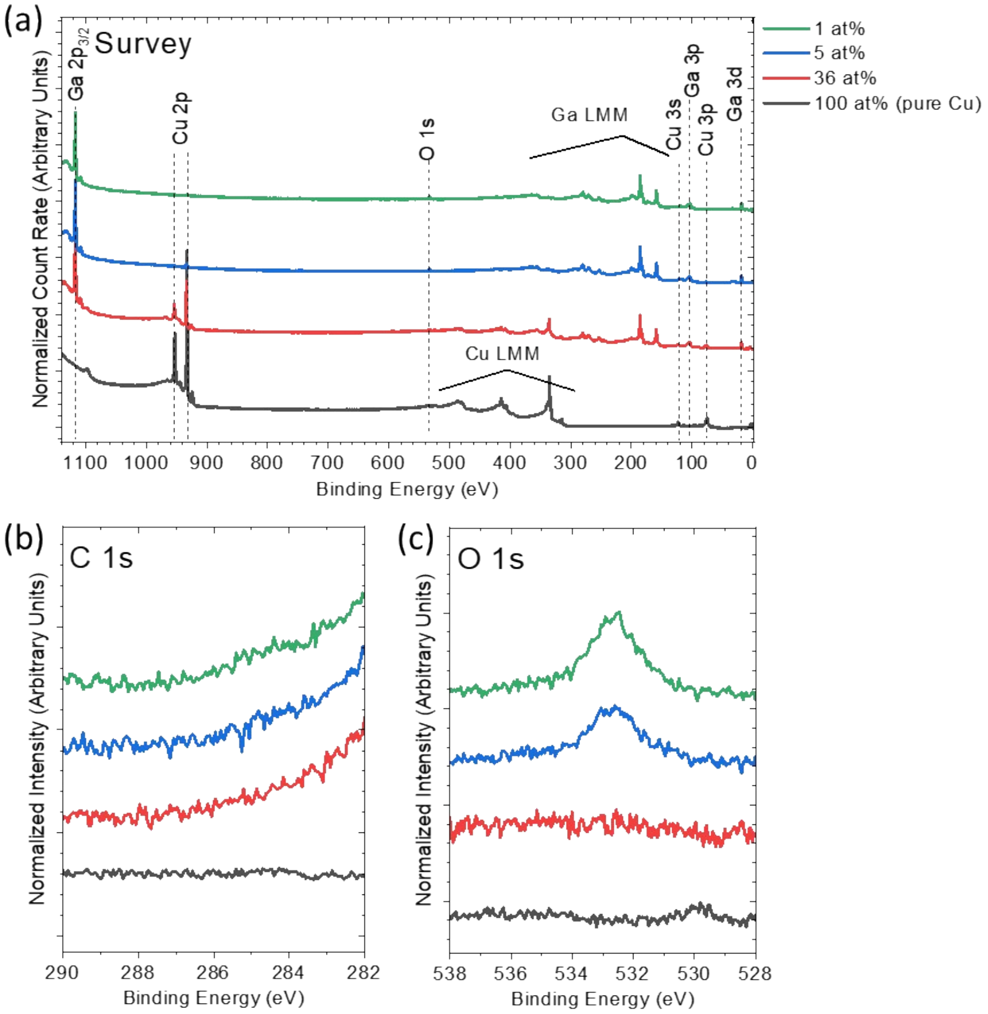

Figure S2. Mg K_α-excited XPS (a) survey spectra, (b) C 1s and (c) O 1s detail spectra of PVD-prepared SiO_x/Si supported Ga-Cu microscopic model system samples with varying Cu contents of 100, 36, 5, and 1 at%. The spectra were measured at room temperature. A vertical constant offset was added to enhance visibility.

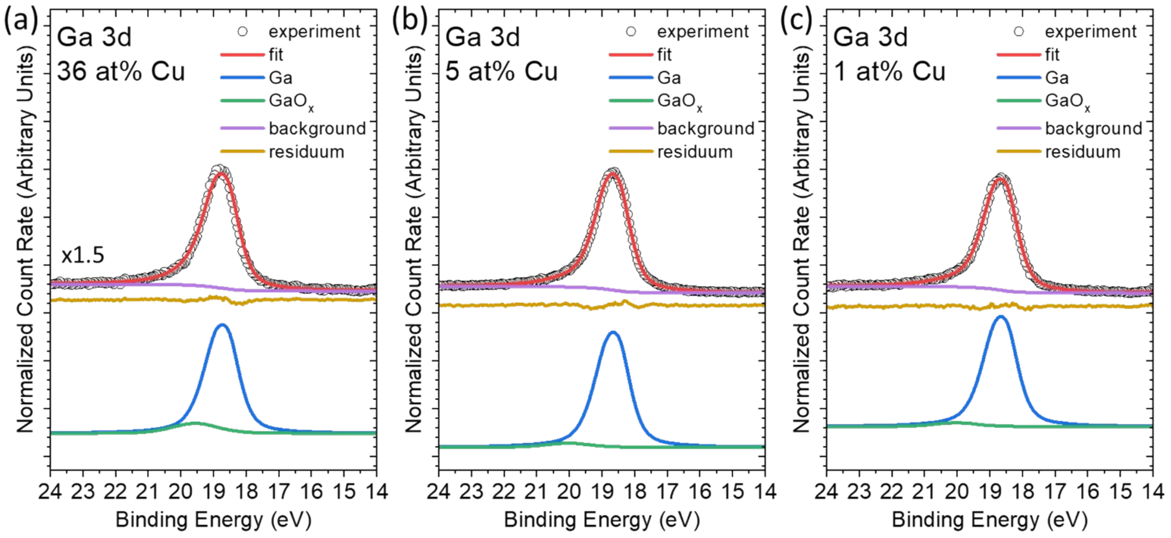

Figure S3. Fit analysis of the Mg K_α excited Ga 3d XPS data of the SiO_x/Si supported Ga-Cu microscopic model system samples with (a) 36 at%, (b) 5 at% and (c) 1 at% Cu contents. The spectra were measured at room temperature. The main metallic Ga peak is fitted by an asymmetric (Doniach-Sunjic) profile, and the minor GaO_x feature is fitted by a Voigt profile. The broad GaO_x peak is expected to represent all various contributions caused by different Ga-O phases and Ga/O compositions (that may result in slightly different E_b values), rather than representing a single species and thus the GaO_x component has not been constraint to have the same peak width or E_b.

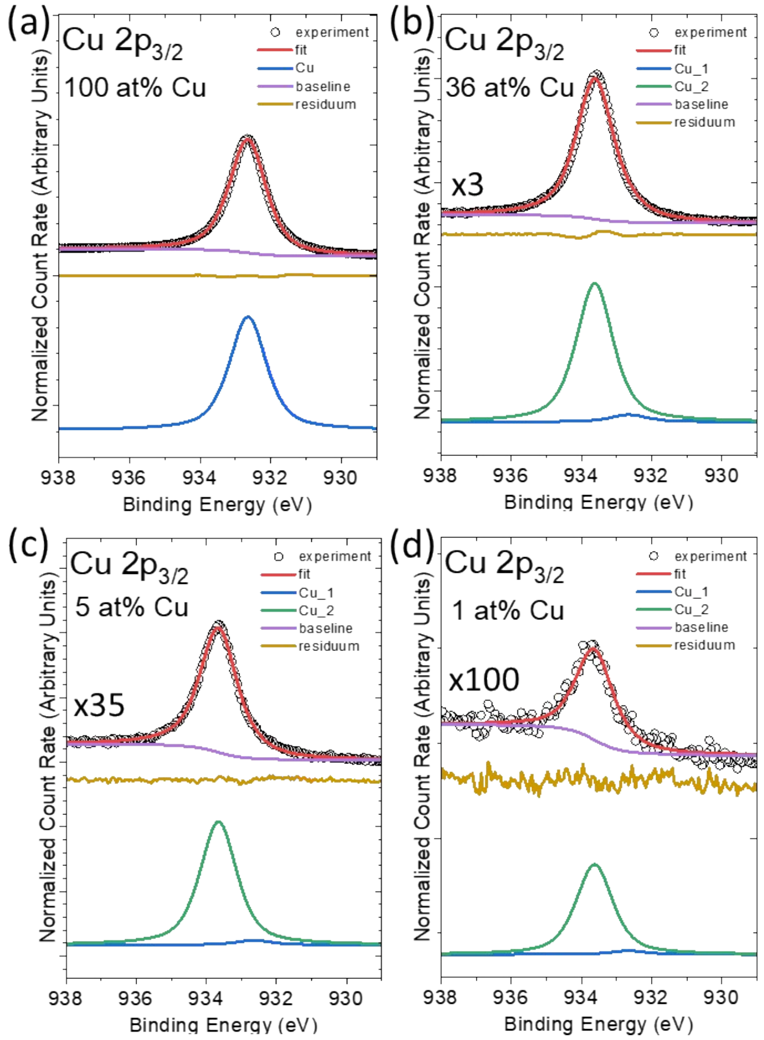

Figure S4. Fit analysis of the Mg K_α excited Cu $2p_{3/2}$ XPS data of the SiO_x/Si supported Ga-Cu microscopic model system samples with (a) 100 at%, (b) 36 at%, (c) 5 at% and (d) 1 at% Cu content. The spectra were measured at room temperature. All (metallic) components are fitted by asymmetric (Doniach-Sunjic) profiles. Note the different magnification factors used to represent the spectra on a similar intensity scale.

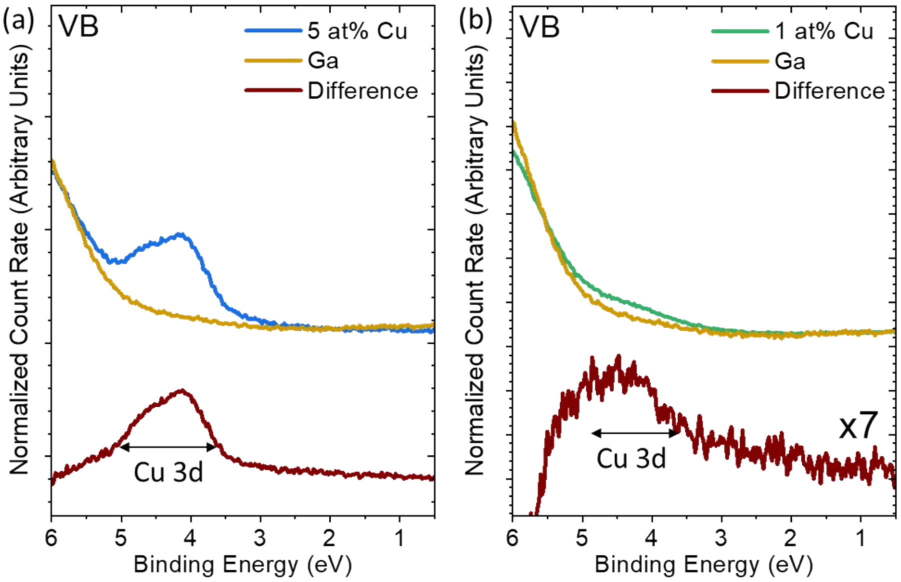

Figure S5. He II-excited UPS valence band (VB) spectra of the SiO_x/Si supported Ga-Cu microscopic model system samples with (a) 5 at% and (b) 1 at% Cu content in direct comparison to that of pure Ga. In addition, the respective difference spectra are shown. The spectra were measured at room temperature.

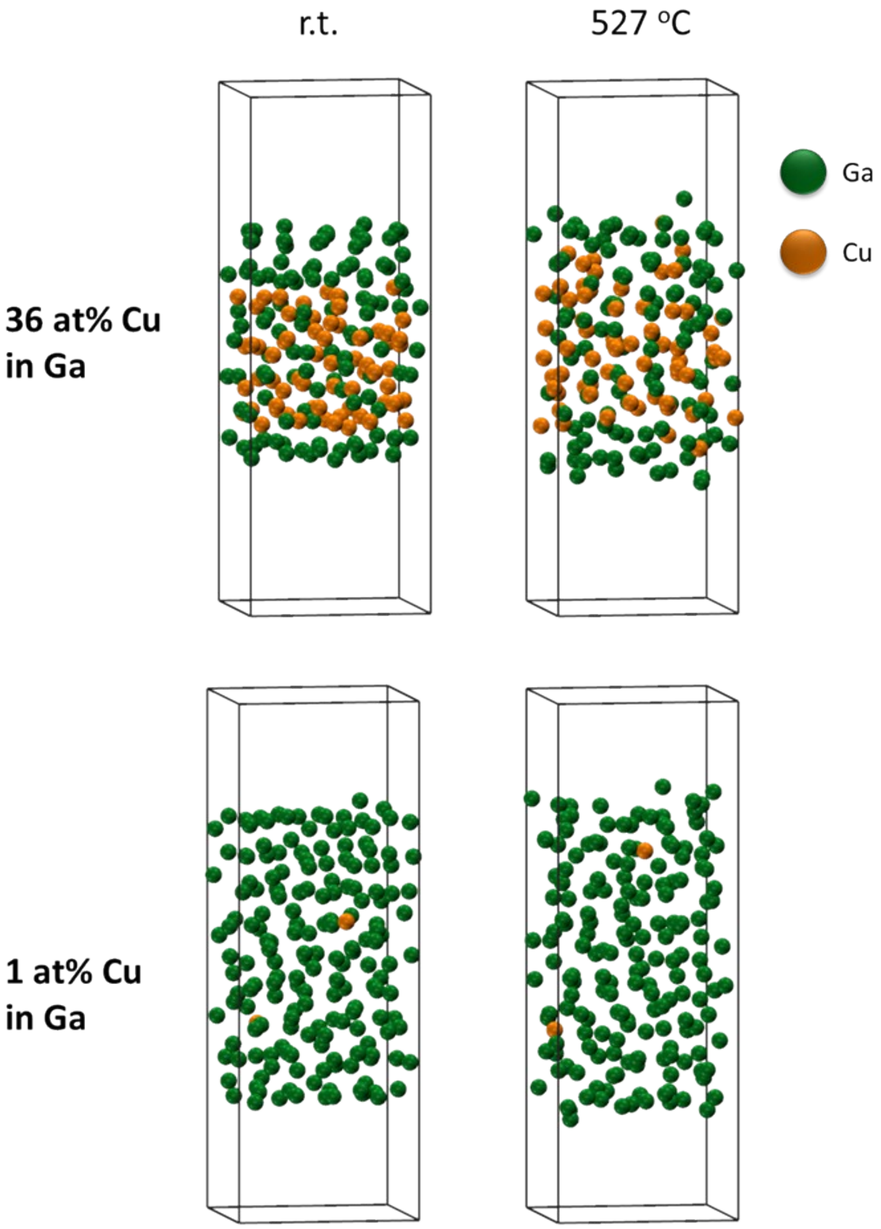

Figure S6. Schematic illustration of the atomic distribution in the calculated Ga-Cu unit cells with 36 at% and 1 at% Cu content derived by ML-FF simulations at r.t. and 527 °C.

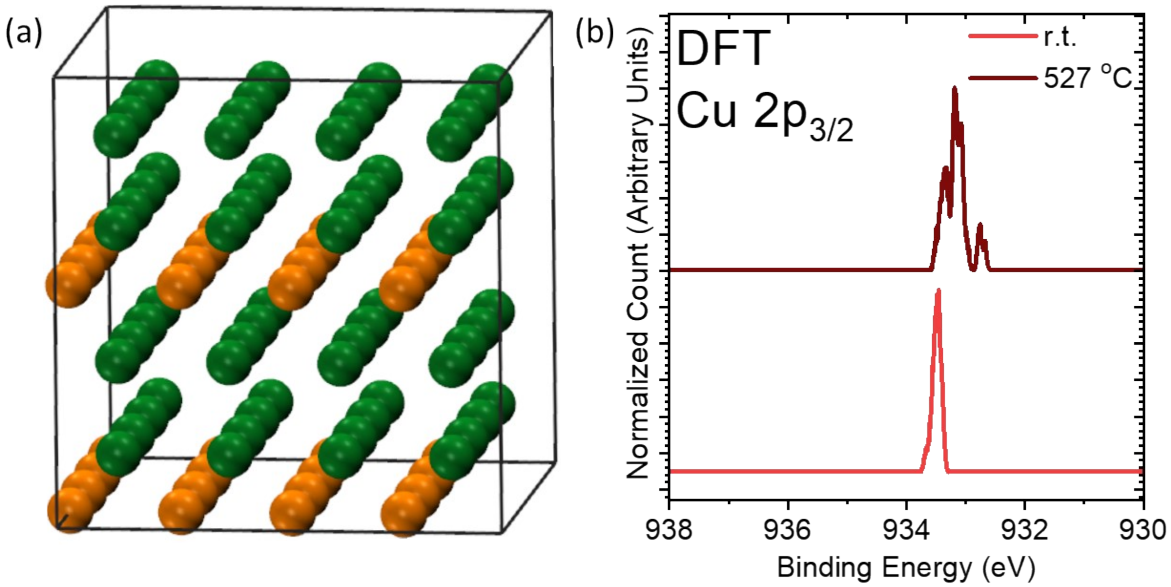

Figure S7. (a) Schematic illustration of the multiplied (4×4×2) and geometry optimized unit cell of Ga_2Cu IMC for the Cu $2p_{3/2}$ core level peak position calculation. (b) DFT-calculated Cu $2p_{3/2}$ core level peak position of Cu atoms in the Ga_2Cu unit cell at r.t. and 527 °C.

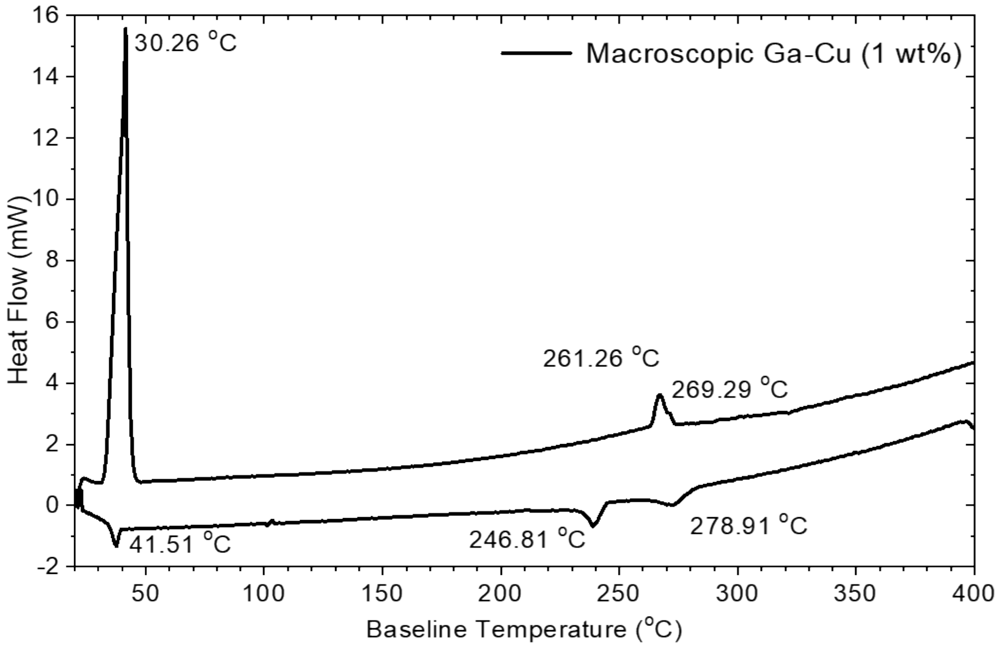

Figure S8. DSC measurement of Ga-Cu bulk sample with 1wt% Cu. During the heating scan, two endothermic peaks were observed - the first peak had an onset temperature of 30.26 °C, corresponding to the melting point of Ga. The onset of the second peak appeared at 261.26 °C, is ascribed to the main melting point, and the shoulder peak at 269.29 °C indicates the complete dissolution of the Ga_2Cu IMC. In the cooling scan, several exothermic peaks were noted. The well-defined peak at 246.81 °C corresponds to the re-crystallization of the Ga_2Cu IMC. The peaks at 41.51 °C and 278.91 °C are attributed to pre-solidification events, with the higher temperature one being associated with the intermetallic compound and the lower temperature one being linked to the solidification of Ga. It is important to note that the re-crystallisation of Ga was not observed. Due to supercooling, freezing of Ga is expected to occur at temperatures below 20 °C.

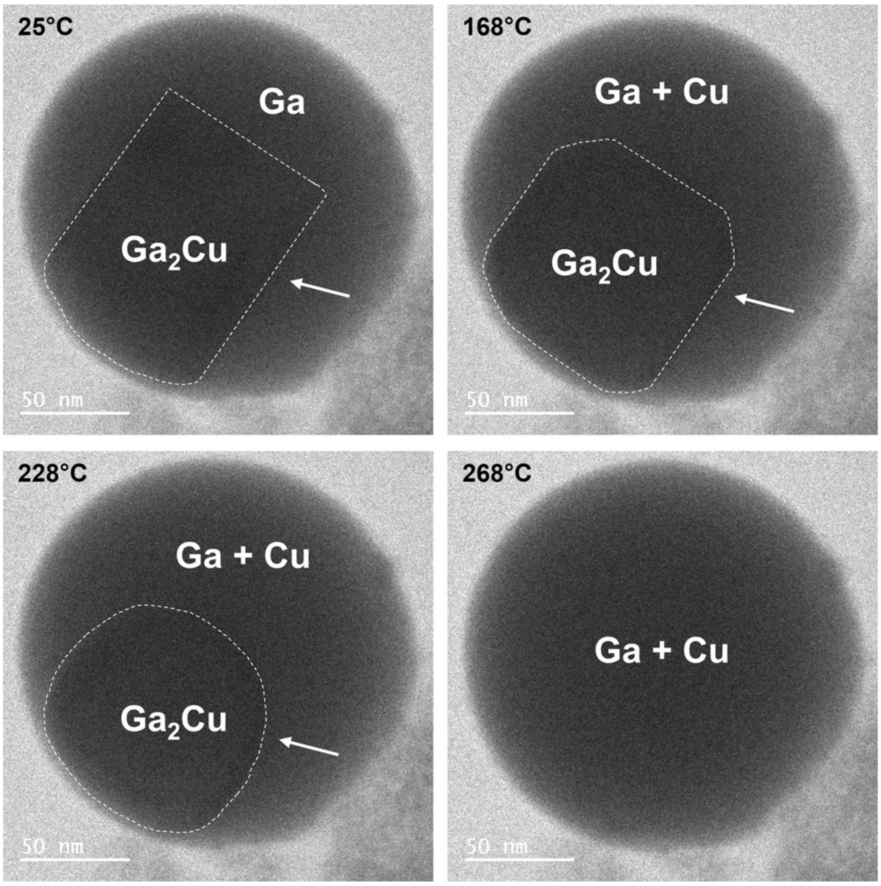

Figure S9. In-situ TEM images of Ga-Cu nanodroplets prepared from a Ga-Cu bulk sample with ≈5 wt% Cu, monitoring the dissolution of the Ga_2Cu IMC upon increasing the temperature from 25°C to 268°C; solid structure is surrounded by dashed line and indicated by an arrow.

Big cell simulation for radial distribution function of Ga-Cu

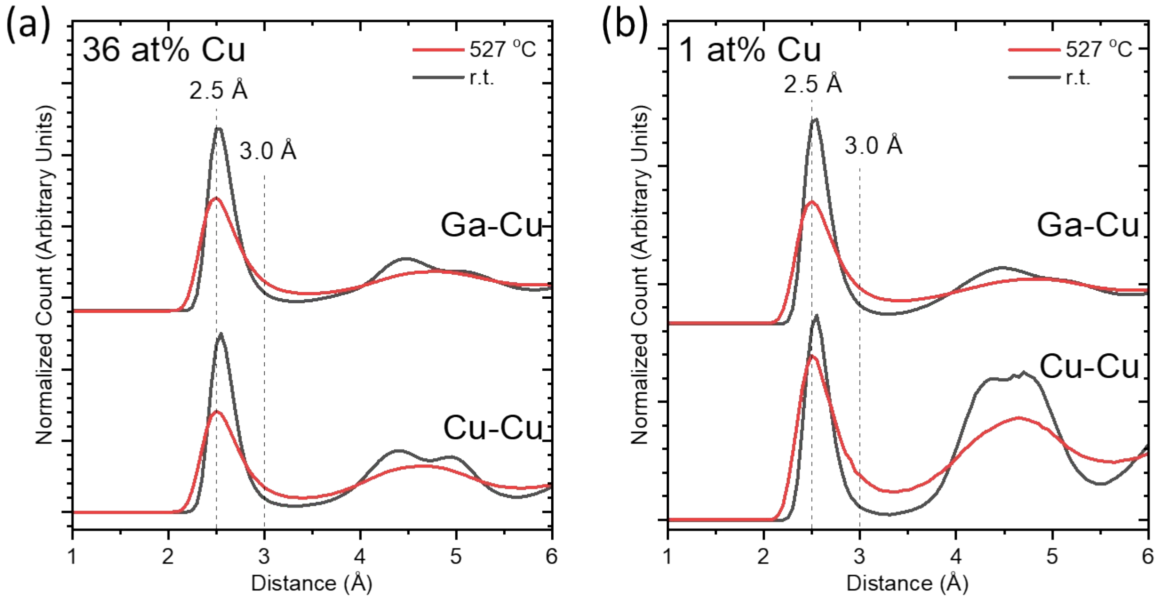

Figure S10. Radial distribution function (RDF) plots of Cu-Cu and Ga-Cu distances in the ML-FF calculated Ga-Cu unit cells with (a) 36 at% and (b) 1 at% Cu content. The calculation is conducted at temperature of 27 °C (r.t.) and 527 °C.

For this simulation a cell with 1200 atoms and the dimensions of 25.3823 × 25.3823 × 60.4587 Å was used. The cell contained 1188 Ga and 12 Cu atoms for the 1% Cu case and 768 Ga and 432 Cu atoms for the 36% Cu system. The respective cell was simulated for 8.000.000 steps of 2 fs length at 300 and 800 K with a previous annealing step consisting of 2.000.000 steps at the respective temperature and the same time step. The radial distribution function is employed to investigate the average bond distance and coordination number of Cu-Cu and Ga-Cu in the ML-FF calculated unit cells with 36 at% and 1 at% Cu content (Fig. S9). To quantify the increase of the Ga-Cu distance, we calculate the normalized count ratio of data points at 2.5 Å and 3.0 Å in the RDF plots:

$$count\ ratio = \frac{I_{2.5\ \text{Å}}}{I_{3.0\ \text{Å}}}$$

For the 36 at% Cu containing unit cell, the count ratio decreases from 12.6 to 3.9; For the 1 at% Cu containing unit cell, the count ratio decreases from 17.2 to 3.6. The decrease of count ratio shows that the radial distribution of the first shell of atoms surrounding a Cu atom expands from 2.5 Å toward 3.0 Å, indicating the extension of average Cu-Cu and Ga-Cu bond distances in the simulated unit cell.

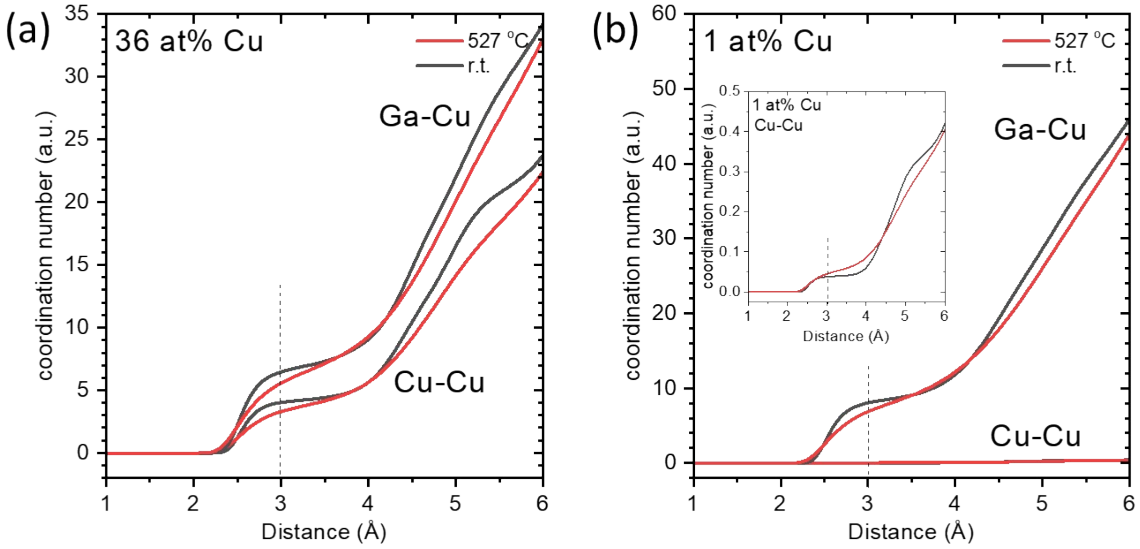

Figure S11. Integrated Cu-Cu and Ga-Cu RDF plots of the ML-FF calculated Ga-Cu unit cells with (a) 36 at% and (b) 1 at% Cu content. The calculation is conducted at temperature of 27 °C (r.t.) and 527 °C.

Furthermore, the integrated radial distribution function (IRDF) demonstrates the local coordination environment of Cu atoms in the shown unit cells (Fig. S10). In the IRDF plot at r.t., we define the coordination number at 3.0 Å as the distance including all adjacent atoms in the first shell, i.e., the total coordination number of 10 and 8 for 36 at% and 1 at% Cu containing unit cells. According to the crystal structure of cubic Cu, each Cu atom is surrounded by 12 Cu atoms.[24] According to the IRDF plots, the coordination number of Cu-Cu bond decreases from 12 to ca. 4 and 0.05 when decreasing the Cu content from 100 at% to 36 at% and 1 at%, respectively. These results suggest a diminishing of the Cu-Cu bond upon Cu dilution. Furthermore, the IRDF plots of both unit cells show a decrease of the Cu-Cu and Ga-Cu

coordination number at 3.0 Å upon 527 °C annealing. The same total coordination number (10 and 8 for 36 at% and 1 at% Cu unit cell) will be reached at ca. 4.0 Å, indicating the annealing induced distance elongation from the central atom to the first shell of atoms.

Furthermore, the Temperature dependent IRDF of Ga_2Cu IMC is also calculated (Fig. S12a). Upon 527 °C annealing, the distribution of both Ga-Cu and Cu-Cu bond length is broadened, which is attributed to the distortion of the unit cell of Ga_2Cu IMC upon annealing, leading to the alternation of Ga-Cu and Cu-Cu bond length and angle (Fig. S12b).

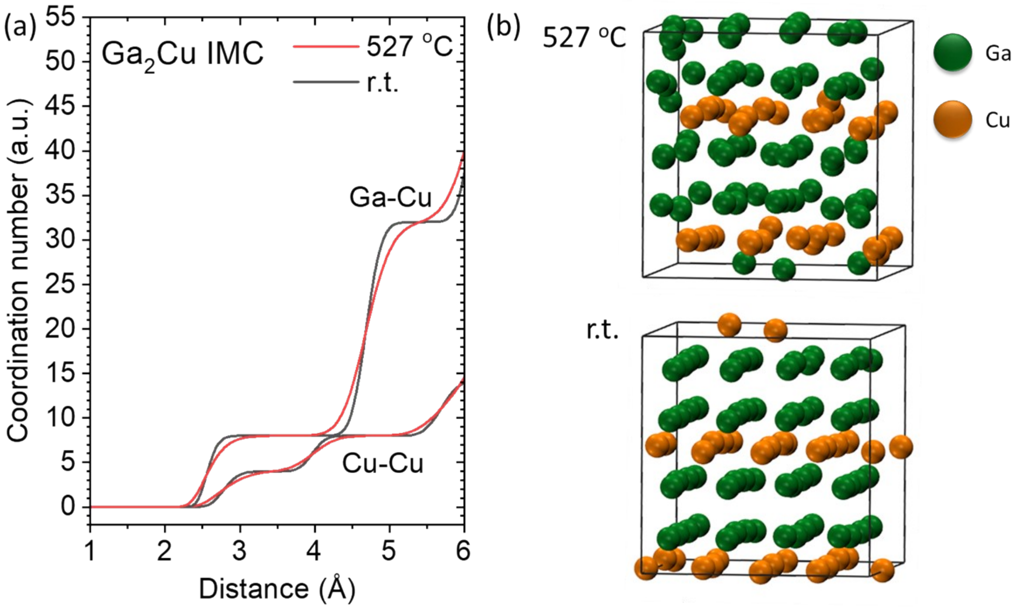

Figure S12. (a) Integrated Cu-Cu and Ga-Cu RDF plots of the ML-FF calculated unit cell of Ga_2Cu IMC. The calculation is conducted at temperature of 27 °C (r.t.) and 527 °C. (b) Schematic illustration of the atomic distribution in the calculated Ga-Cu unit cells of Ga_2Cu IMC derived by ML-FF simulations at r.t. and 527 °C after 100000 steps.

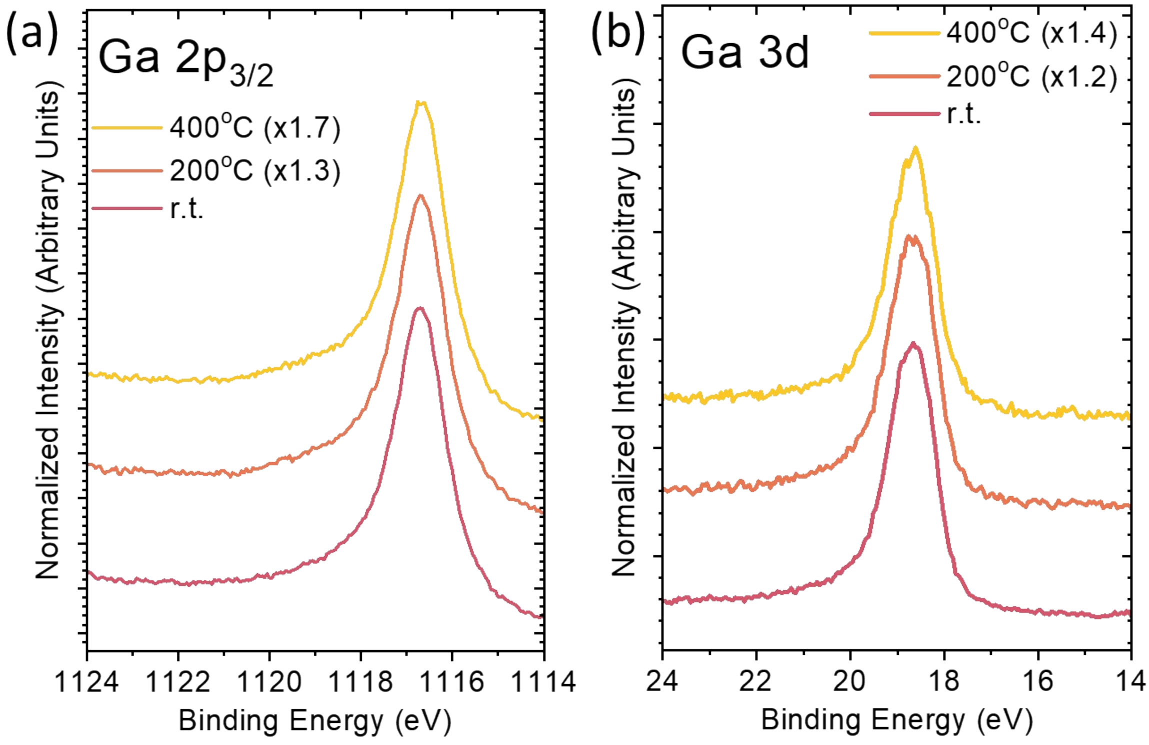

Figure S13. Mg K_α-excited (a) Ga $2p_{3/2}$ and (b) Ga 3d XPS detail spectra of the SiO_x/Si supported Ga-Cu microscopic model samples containing 1 at% Cu measured at r.t., 200 °C, and 400 °C. Note the magnification factors for the data measured at elevated temperatures indicating an intensity decrease. A vertical constant offset was added to enhance visibility.

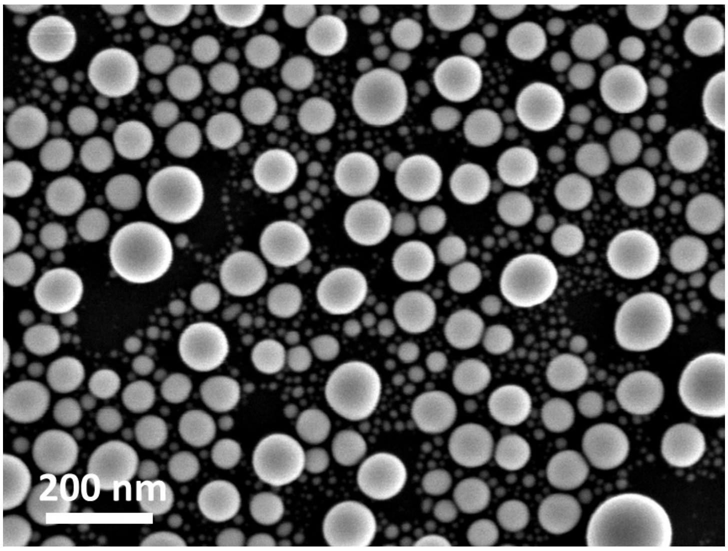

Figure S14. Representative SEM image of the SiO_x/Si supported Ga-Cu microscopic model system sample with 1 at% Cu on SiO_x/Si support after 400 ^{o}C annealing in UHV condition, i.e., $\leq 1x10^{-8}$ mbar.

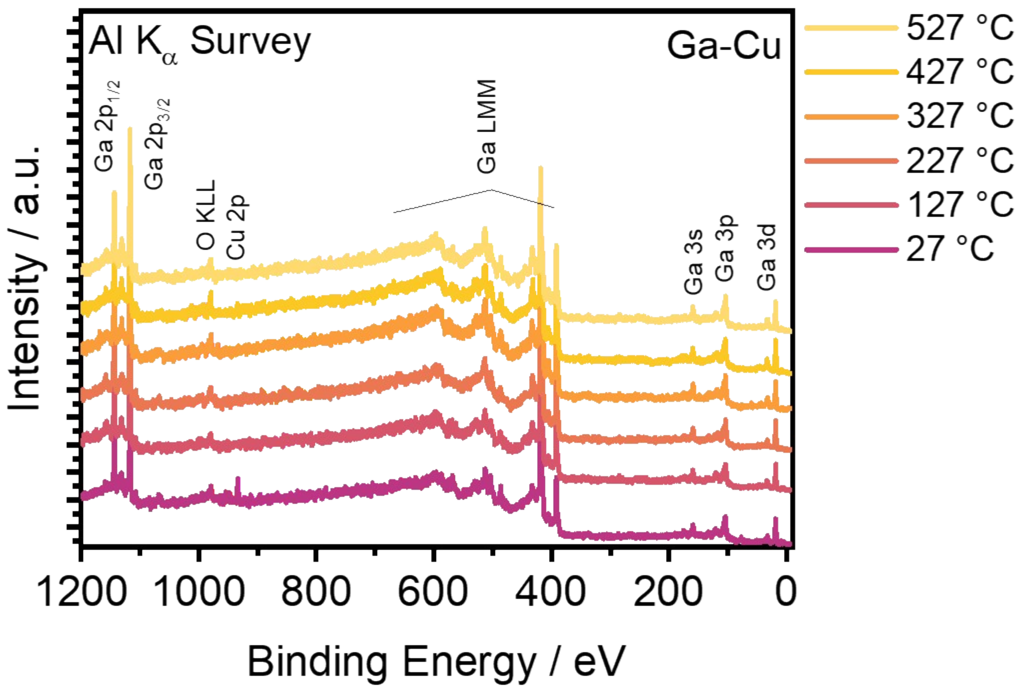

Figure S15. Al K_α-excited temperature dependent XPS survey spectra of the Ga-Cu macroscopic model system samples with 1 at% Cu content measured at different temperatures. A vertical constant offset was added to enhance visibility.

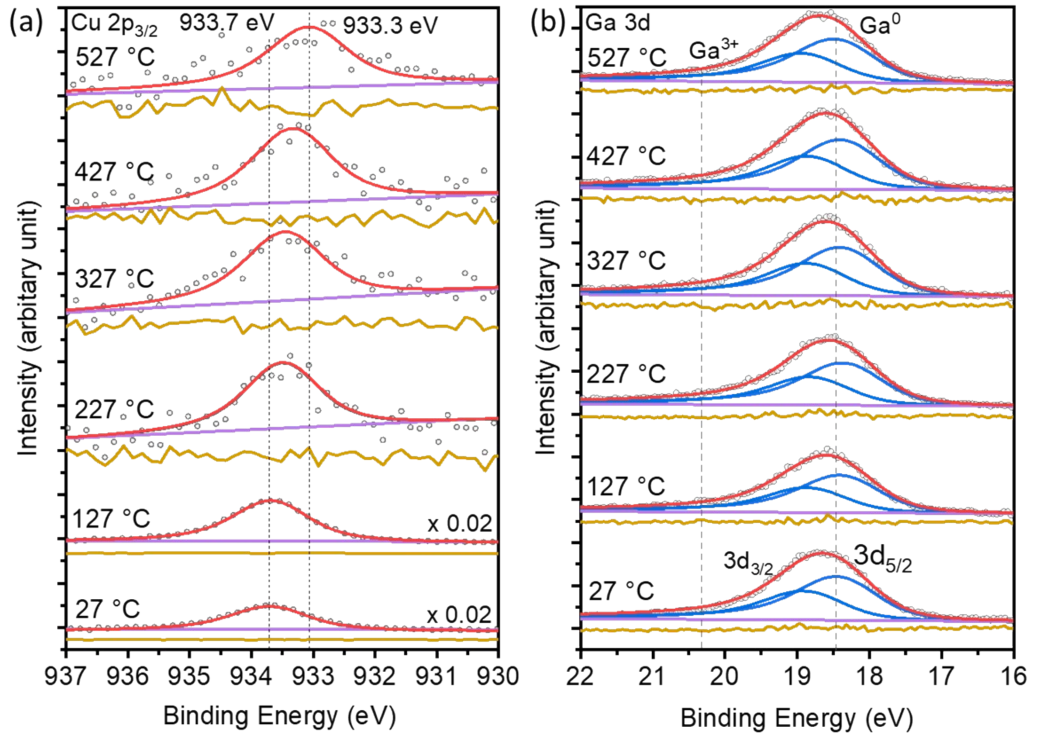

Figure S16. Temperature dependent XPS spectra and fit analysis of the Ga-Cu macroscopic model system sample with 1 at% Cu content. A vertical constant offset was added to enhance visibility. The vertical lines indicate the Cu $2p_{3/2}$ and $Ga3d_{5/2}$ E_b position at r.t. for easy tracking of temperature induced changes. All residue lines in (a) are scaled by 0.5x for the better visibility of the stacked spectra.

Ga-Ag and Ga-Au model system samples: Detailed data discussion

To further establish the fundamental properties of Ga – filled d-band TM binary alloys, XPS measurements of Ga-Ag and Ga-Au based microscopic and macroscopic model systems have been conducted and will be discussed below. In the microscopic Ga-Ag and Ga-Au system, a TM core level peak shift to higher E_b with respect to the metallic references is observed (Fig. S17). The corresponding survey spectra of the two microscopic model systems are shown in Fig. S18 and S19, indicating, a high-quality sample surface with only minor amounts of oxygen and carbon present, which is similar to the Ga-Cu system discussed in detail in the main text. Correspondingly, we find the Ga matrix to be metallic, as also documented by the Ga $2p_{3/2}$ spectra shown in Fig. S20.

Fig. S17a shows the Ag $3d_{5/2}$ region for a pure Ag sample and a microscopic Ga-Ag model system sample with 1 at% Ag content prepared by PVD. For the pure Ag sample (100 at% Ag), the Ag $3d_{5/2}$ line is observed at 368.2 eV, which can be attributed to metallic Ag. Upon decreasing the Ag concentration to 1 at%, a +0.7 eV E_b shift to higher E_b is observed, which is attributed to the formation of a Ga-Ag alloy with significantly decreased Ag-Ag interaction. The detailed spectral fitting results of the Ag $3d_{5/2}$ spectra are shown in Fig. S21. Upon Ag dilution, the signal of metallic Ag is significantly decreased and a new species noted as Ga-Ag alloy is formed.

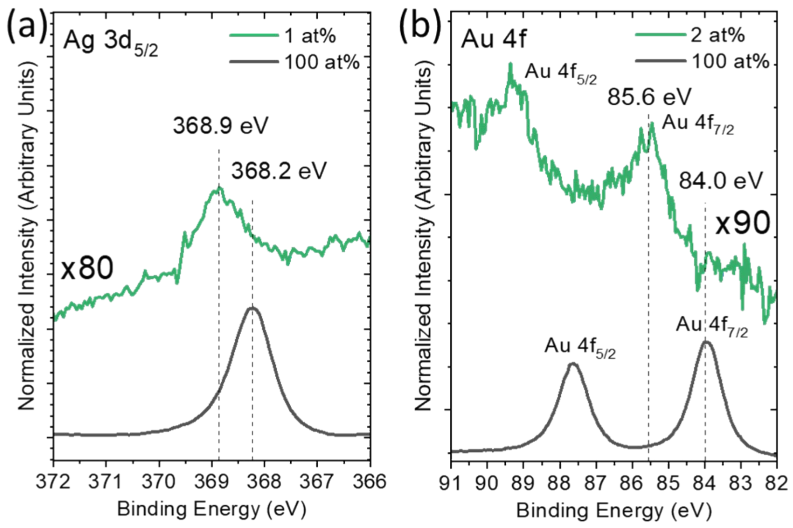

Figure S17. Mg K_α-excited XPS detail spectra of SiO_x/Si supported (a) Ga-Ag (Ag $3d_{5/2}$) and (b) Ga-Au (Au 4f) microscopic model system and reference samples with 100 at% and 2 at% Ag as well as 100 at% and 1 at% Au content, respectively. The spectra are measured at room temperature. A vertical constant offset was added to enhance visibility.

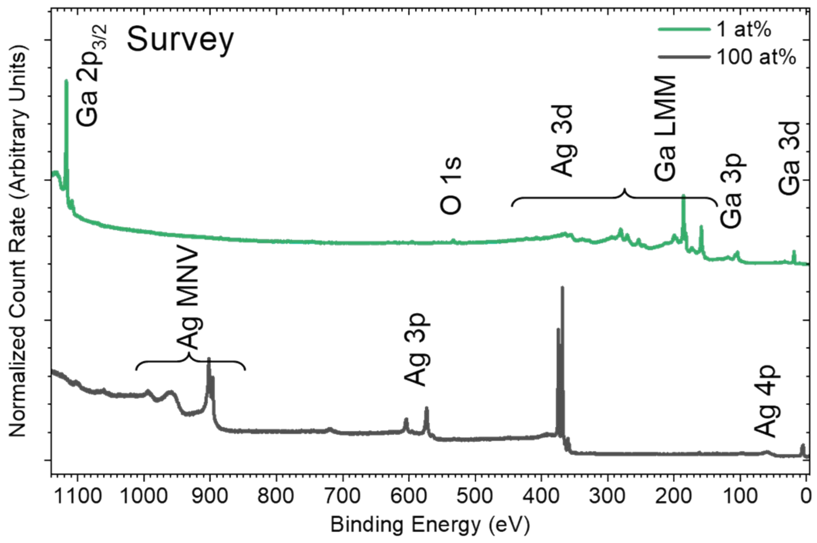

Figure S18. Mg K_α-excited XPS survey spectra of the PVD-prepared SiO_x/Si supported Ga-Ag microscopic model system samples with 100 and 1 at% Ag content. The spectra are measured at room temperature. A vertical constant offset was added to enhance visibility.

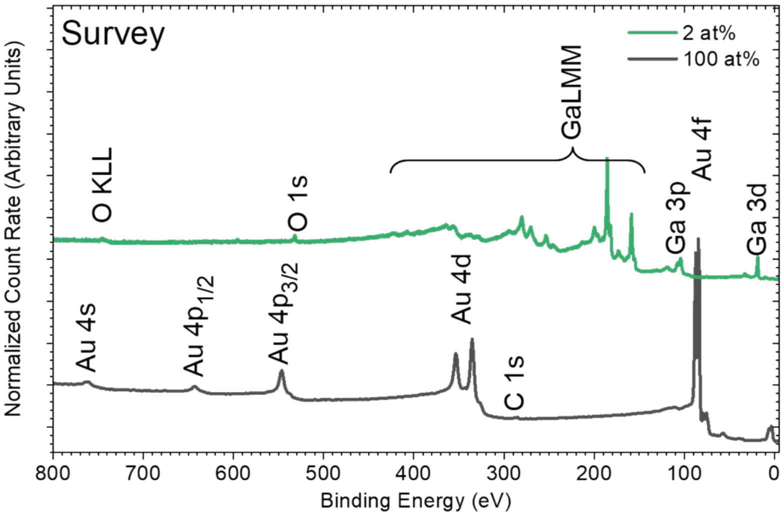

Figure S19. Mg K_α-excited XPS survey spectra of sputter-cleaned Au foil (100 at%) and a PVD-prepared SiO_x/Si supported Ga-Au microscopic model system sample with 2 at% Au content. The spectra are measured at room temperature. A vertical constant offset was added to enhance visibility.

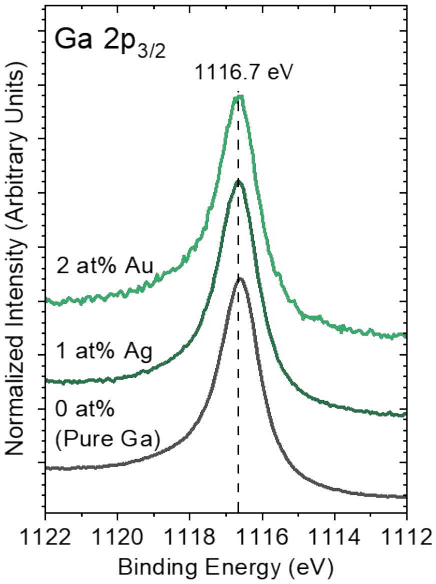

Figure S20. Mg K_α-excited XPS Ga $2p_{3/2}$ detail spectra of the PVD-prepared SiO_x/Si supported Ga-Ag and Ga-Au microscopic model system samples with 1 at% Ag and 2 at% Au content. The spectrum of PVD-prepared pure Ga (0 at%) is plotted as reference. The spectra are measured at room temperature. A vertical constant offset was added to enhance visibility. The vertical line at 1116.7 eV E_b indicates the peak position of metallic Ga.[25]

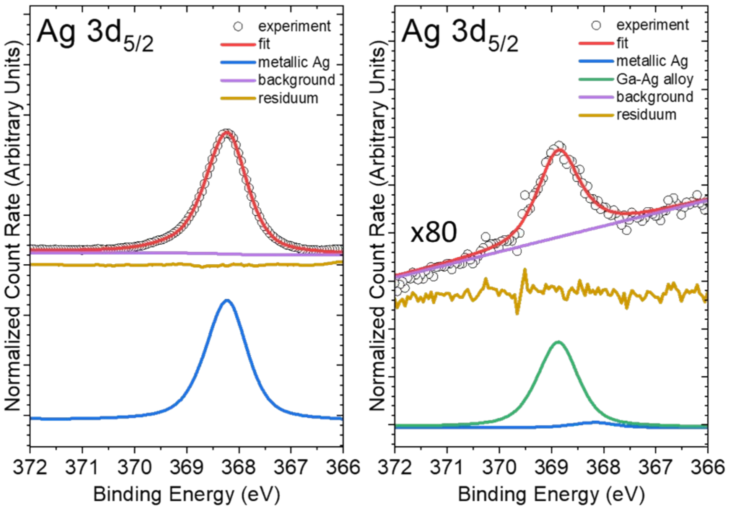

Figure S21. Fit analysis of the Mg K_α excited Ag $3d_{5/2}$ XPS detail spectra of (a) a PVD-prepared Ag thin film ("metallic Ag") and (b) a SiO_x/Si supported Ga-Ag microscopic model system sample with 1 at% Ag. The spectra are measured at room temperature. A vertical constant offset was added to enhance visibility.

Fig. S17b shows the Au 4f region of pure Au (100 at% Au) and a Ga-Au microscopic model system sample with 2 at% Au content. The Au $4f_{7/2}$ line of the pure Au reference is found at 84.0 eV with a Au $4f_{5/2}$ – Au $4f_{7/2}$ spin-orbit split of 3.7 eV, in agreement with the literature.[26] For the 2 at% Au sample of the Ga-Au microscopic model system, a 1.6 eV shift of the Au $4f_{7/2}$ core level peak to higher E_b is observed (Fig. S17b), which can be attributed to the Ga-Au alloying. Fit analysis of the Au $4f_{7/2}$ spectrum of the Au reference suggests the presence of an additional high E_b component, which we ascribe to the presence of Au-O bonds attributed to the residual Au-oxide on the sputter-cleaned Au foil which has been stored in ambient conditions before the experiments (Fig. S22a). For the 2 at% Au sample of the Ga-Au microscopic model system, a component with 0.4 eV wider peak width is used for the fitting shown in Fig. S22b, indicating a less ordered chemical environment with respect to the Au reference with different bond angles and lengths.

In summary for both the Ga-Ag and Ga-Au microscopic model system samples, we find the TM core level line to be shifted to higher E_b which is attributed to the formation of alloy species with isolated TM atoms. This E_b shift is in good agreement with the observation in the Ga-Cu system, proving that the alloying of filled d-band transition metals with Ga is possible and that the resulting core level peak shift to higher E_b can be used as an indication for site isolation.

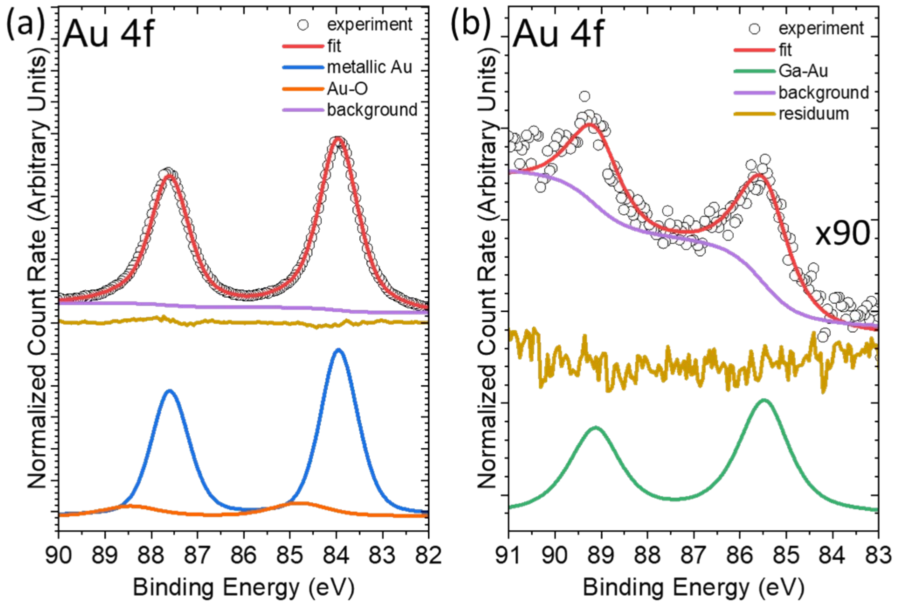

Figure S22. Fit analysis of Mg K_α excited Au 4f XPS detail spectra of (a) a sputter-cleaned Au foil and (b) a SiO_x/Si supported Ga-Au microscopic model system sample with 2 at% Au. The spectra are measured at room temperature. The wider spectral width of the fit component in (b) is attributed to the formed Ga-Au alloy being less ordered than the Au reference, resulting in a material with different bond angles and distances. The Au-O feature in (a) is attributed to residual Au-oxide on the sputter-cleaned Au foil which has been stored in ambient conditions before the experiments.

Further, XPS measurements on Ga-Ag and Ga-Au macroscopic models system samples with 1 at% TM content were conducted to elucidate the temperature-dependent E_b evolution and surface phase behavior of these binary alloys (Fig. S23). The temperature-dependent Ag $3d_{5/2}$ and Au $4f_{7/2}$ core level shifts for the Ga-Ag and Ga-Au systems are summarized in Fig. S23a, b. The corresponding core level spectra and survey spectra are shown in Fig. S24-S27.

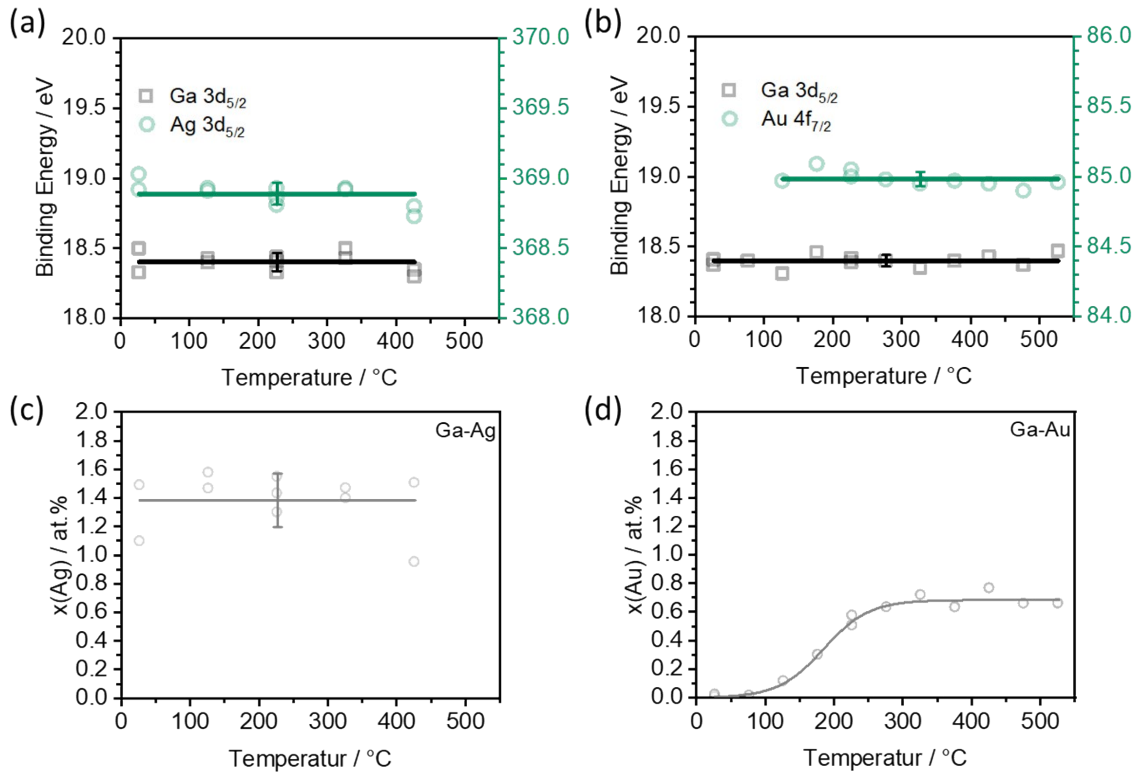

Figure S23. Summary of temperature-dependent Al K_α excited XPS measurements of the Ga-TM macroscopic model system samples with nominal 1 at% Ag and 1 at% Au content. Panels (a, b) show the Ag $3d_{5/2}$, Au $4f_{7/2,}$ and Ga 3d core level peak E_b positions as a function of temperature. Panels (c, d) show the temperature-dependent variation of the XPS-derived Ag and Au surface content.

In Fig. S23a, the Ag $3d_{5/2}$ and Ga $3d_{5/2}$ E_b changes as a function of increasing temperature of the Ga-Ag macroscopic model systems are shown. At low temperatures, the E_b of the Ag $3d_{5/2}$ line is 368.9 eV and in good agreement with the results of the Ga-Ag microscopic model system sample discussed in conjunction with Fig. S17. The Ga $3d_{5/2}$ component is found at 18.4 eV. The signals do not shift with increasing temperature, indicating a temperature-independent

chemical environment of Ag in Ga. This deviates from the temperature-induced E_b shift observed for the Ga-Cu model system. However, a comparison with the phase diagram shows that a melting point of 30 °C is expected for the Ga-Ag alloy and a supercooling of liquid Ga alloys is expected.[27] Hence, we interpret the constant E_b as an indication for the Ga-Ag samples to be present in a liquid state right from the start of the experiment.

Fig. S23b shows the evolution of the Au $4f_{7/2}$ and Ga $3d_{5/2}$ E_b of the Ga-Au macroscopic models system sample with increasing temperature. The Au 4f signal was not observed at temperatures below 77 °C. The Au $4f_{7/2}$ level is found at E_b of 85.0 eV starting from 127 °C and the Ga $3d_{5/2}$ is found at 18.4 eV at low temperatures, which can be attributed to the presence of Ga-Au alloys.[28] The signals show no change throughout the experiment, indicating again a temperature-independent chemical environment of Au in Ga at the surface. In Fig. S23c, the XPS-derived Ag surface content is plotted as a function of sample temperature of a Ga-Ag macroscopic model system sample with a nominal Ag content of 1 at%. Between 27 and 427 °C, the surface Ag content stays constant at 1.4 ± 0.2 at.%. A comparison with the phase diagram shows that a melting point of 30 °C is expected for the Ga-Ag alloy.[27] As also no E_b shift was observed (Fig. S23a), we interpret the results as the alloy being liquid throughout the experiment. The calculated content of 1.4 at% indicates a significantly Ag-enriched surface compared to the nominal bulk content of 1 at.%.

The evolution of the XPS-derived Au surface concentration of a Ga-Au macroscopic model system sample with a nominal Au content of 1 at% with increasing temperature resembles the behaviour of other TMs, i.e., Rh, Pd, and Pt, in Ga found in earlier studies (Fig. S23d).[1, 29-30] At 27 and 77 °C, no significant Au 4f signal was found, as the surface was (at least withing the detection limit of XPS) completely void of any Au. Here, Au-rich phases segregating to the bulk of the sample, thereby depleting Au from the surface, could be an explanation. At higher temperatures, the amount of Au in the surface-near region steadily increases until it reaches 0.7

± 0.1 at.%. This concentration remains constant between 327 and 527 °C. Considering the Ga-Au phase diagram, indicating a melting of the Au-rich phase at 57°C,[36] we interpret this evolution as indication that the Au-rich phase segregated to the bulk of the sample gradually melts. The released Au is mobile and disperses in the liquid alloy, thereby increasing the surface content until a plateau of 0.7 ± 0.1 at% Au is reached. However, a successful alloying of Ag and Au with Ga is observed below typical reaction temperatures, e.g., 500 °C.

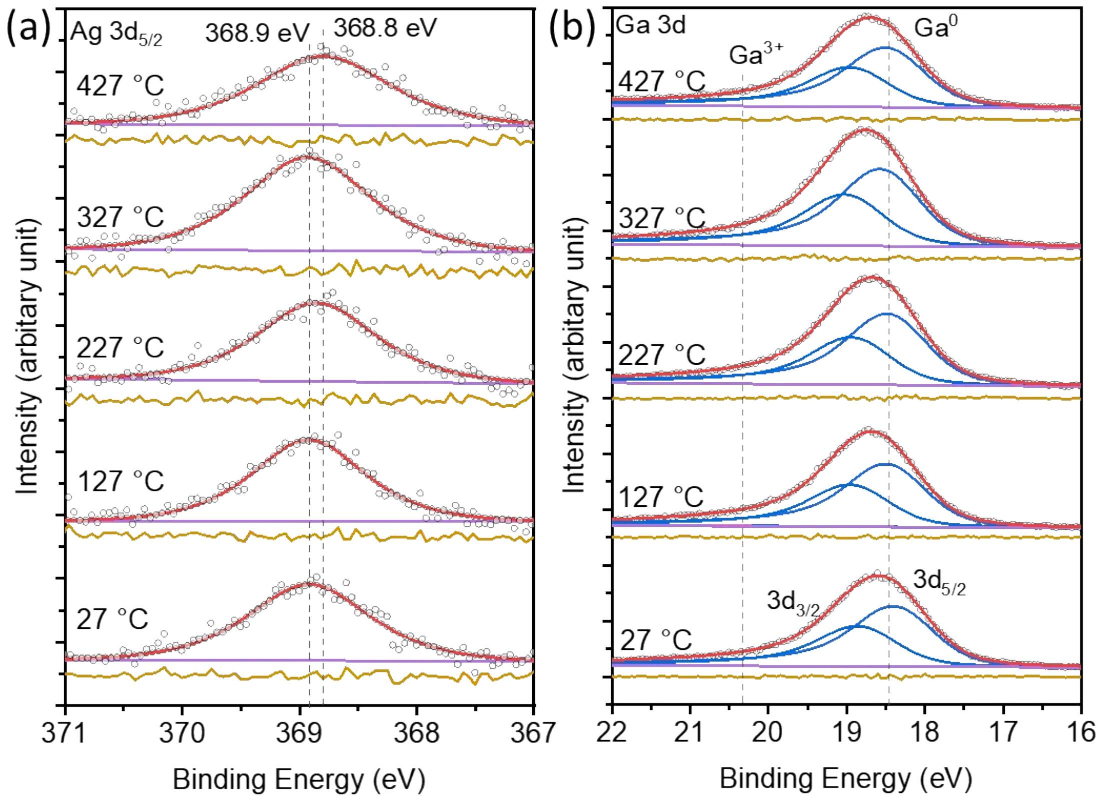

Figure S24. Temperature dependent XPS data of (a) Ag $3d_{5/2}$ and (b) Ga 3d core level lines of a Ga-Ag macroscopic model system sample with a nominal Ag content of 1 at%. The measurements are conducted with Al K_α X-ray (1486.58 eV). A vertical constant offset was added to enhance visibility. The vertical lines indicate the Ag $3d_{5/2}$ and Ga$3d_{5/2}$ E_b position, making it easier to identify spectral changes upon annealing. All residue lines in (a) are scaled by 0.5x for the better visibility of the stacked spectra.

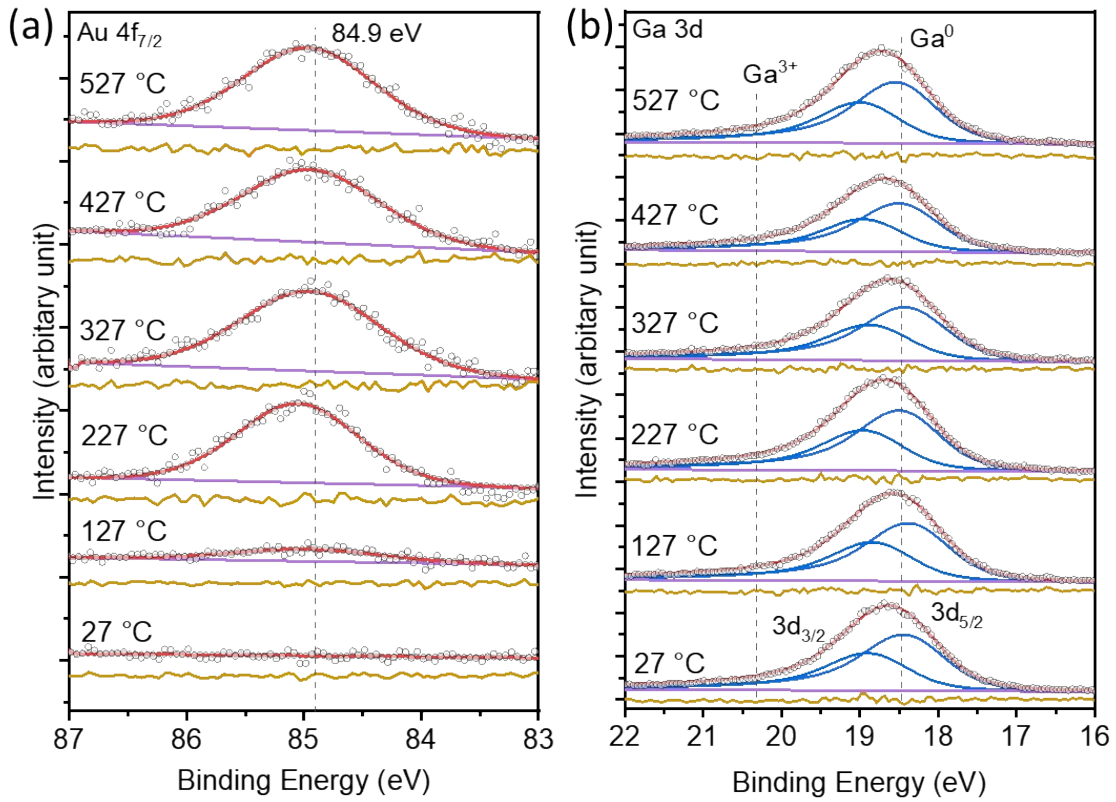

Figure S25. Temperature dependent XPS data of (a) Au $4f_{7/2}$ and (b) Ga 3d core level lines of a Ga-Ag macroscopic model system sample with a nominal Ag content of 1 at%. The measurements are conducted with Al K_{α} X-ray (1486.58 eV). A vertical constant offset was added to enhance visibility. The vertical lines indicate the Ag $3d_{5/2}$ and Ga$3d_{5/2}$ E_b positions, making it easier to identify spectral changes upon annealing. All residue lines in (a) are scaled by 0.5x for the better visibility of the stacked spectra.

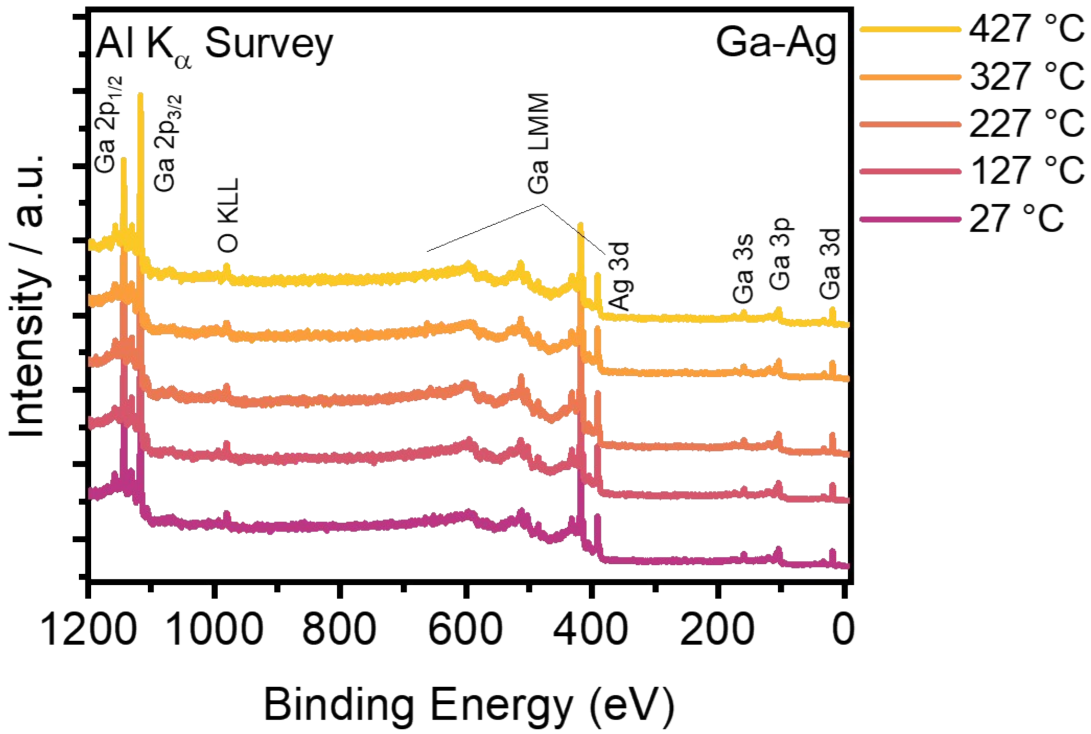

Figure S26. Al K_{α}-excited temperature dependent XPS survey spectra of a Ga-Ag macroscopic model system sample with 1 at% Ag content measured at different temperatures. A vertical constant offset was added to enhance visibility.

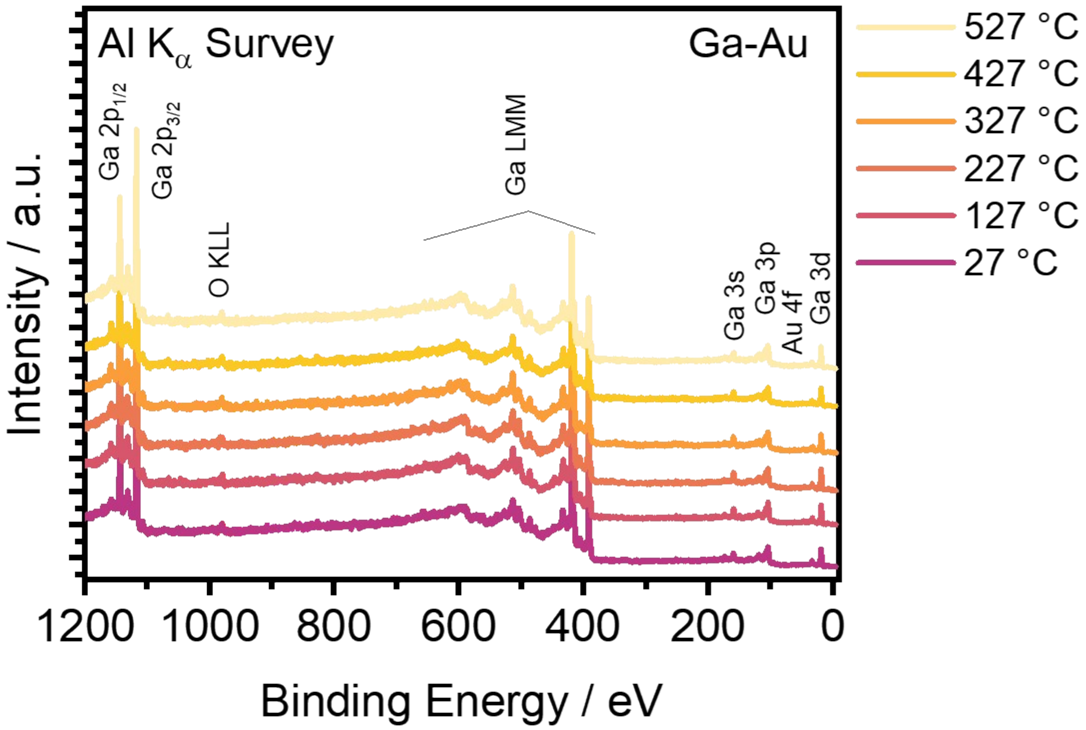

Figure S27. Al K_{α}-excited temperature dependent XPS survey spectra of the Ga-Au macroscopic model system with 1 at% Au content measured at different temperatures. A vertical constant offset was added to enhance visibility.

Table S1. TEM derived Ga_2Cu IMC dissolution for a Ga-Cu nanodroplet prepared from a Ga-Cu bulk sample with ≈10 wt% Cu. Complete dissolution was observed at 310°C.

Temperature (°C)	Dissolved (wt%)
25	0
170	1
200	1.3
230	2.9
240	3.3
250	4.9
260	6
270	7.5
280	6.6
290	7.5
300	8.7
310	9.6
320	9.1

References

1. Grabau, M.; Krick Calderón, S.; Rietzler, F.; Niedermaier, I.; Taccardi, N.; Wasserscheid, P.; Maier, F.; Steinrück, H.-P.; Papp, C., Surface Enrichment of Pt in Ga2O3 Films Grown on Liquid Pt/Ga Alloys. *Surf. Sci.* **2016,** *651*, 16-21.

2. Pantförder, J.; Pöllmann, S.; Zhu, J. F.; Borgmann, D.; Denecke, R.; Steinrück, H. P., New setup for in situ x-ray photoelectron spectroscopy from ultrahigh vacuum to 1mbar. *Rev. Sci. Instrum.* **2004,** *76*, 014102.

3. Gengenbach, T. R.; Major, G. H.; Linford, M. R.; Easton, C. D., Practical guides for x-ray photoelectron spectroscopy (XPS): Interpreting the carbon 1s spectrum. *J. Vac. Sci. Technol., A* **2021,** *39*, 013204.

4. Trzhaskovskaya, M. B.; Nikulin, V. K.; Nefedov, V. I.; Yarzhemsky, V. G., Non-dipole second order parameters of the photoelectron angular distribution for elements Z=1–100 in the photoelectron energy range 1–10keV. *At. Data Nucl. Data Tables* **2006,** *92*, 245-304.

5. Trzhaskovskaya, M. B.; Nikulin, V. K.; Nefedov, V. I.; Yarzhemsky, V. G., Influence of Nondipolar Effects on The Photoelectron Angular Distribution upon Photoionization of 2p and 3d Atomic Shells. *Opt. Spectrosc.* **2004,** *96*, 765-773.

6. Yeh, J. J.; Lindau, I., Atomic subshell photoionization cross sections and asymmetry parameters: $1 \leqslant Z \leqslant 103$. *At. Data Nucl. Data Tables* **1985,** *32*, 1-155.

7. Shinotsuka, H.; Tanuma, S.; Powell, C. J.; Penn, D. R., Calculations of Electron Inelastic Mean Free Paths. XII. Data for 42 Inorganic Compounds over The 50 eV to 200 keV Range with The Full Penn Algorithm. *Surf. Interface Anal.* **2019,** *51*, 427-457.

8. Shinotsuka, H.; Da, B.; Tanuma, S.; Yoshikawa, H.; Powell, C. J.; Penn, D. R., Calculations of Electron Inelastic Mean Free Paths. XI. Data for Liquid Water for Energies from 50 eV to 30 keV. *Surf. Interface Anal.* **2017,** *49*, 238-252.

9. Shinotsuka, H.; Tanuma, S.; Powell, C. J.; Penn, D. R., Calculations of Electron Inelastic Mean Free Paths. X. Data for 41 Elemental Solids over The 50 eV to 200 keV Range with The Relativistic Full Penn Algorithm. *Surf. Interface Anal.* **2015,** *47*, 1132-1132.

10. Wolfgang, W.; Werner, S.; Cedric, P.; Justin, G., Simulation of Electron Spectra for Surface Analysis (SESSA)Version 2.2 User's Guide. Natl Std. Ref. Data Series (NIST NSRDS), National Institute of Standards and Technology, Gaithersburg, MD: 2021.

11. Kresse, G.; Furthmüller, J., Efficient Iterative Schemes for Ab Initio Total-Energy Calculations Using a Plane-Wave Basis Set. *Phys. Rev. B* **1996,** *54*, 11169-11186.

12. Kresse, G.; Furthmüller, J., Efficiency of Ab-Initio Total Energy Calculations for Metals and Semiconductors Using a Plane-Wave Basis Set. *Comput. Mater. Sci.* **1996,** *6*, 15-50.

13. Jinnouchi, R.; Karsai, F.; Kresse, G., On-the-fly machine learning force field generation: Application to melting points. *Phys. Rev. B* **2019,** *100*, 014105.

14. Perdew, J. P.; Burke, K.; Ernzerhof, M., Generalized Gradient Approximation Made Simple. *Phys. Rev. Lett.* **1996,** *77*, 3865-3868.

15. Methfessel, M.; Paxton, A. T., High-Precision Sampling for Brillouin-Zone Integration in Metals. *Phys. Rev. B* **1989,** *40*, 3616-3621.

16. Grimme, S.; Antony, J.; Ehrlich, S.; Krieg, H., A consistent and accurate ab initio parametrization of density functional dispersion correction (DFT-D) for the 94 elements H-Pu. *J. Chem. Phys.* **2010,** *132*, 154104.

17. Andersen, H. C., Molecular dynamics simulations at constant pressure and/or temperature. *J. Chem. Phys.* **1980,** *72*, 2384-2393.

18. Nosé, S., A Unified Formulation of the Constant Temperature Molecular Dynamics Methods. *J. Chem. Phys.* **1984,** *81*, 511-519.

19. Hoover, W. G., Canonical dynamics: Equilibrium phase-space distributions. *Phys. Rev. A* **1985,** *31*, 1695-1697.

20. Steffen, J., Utility scripts and programs for VASP calculations. https://github.com/Trebonius91/utils4VASP.

21. Pueyo Bellafont, N.; Viñes, F.; Hieringer, W.; Illas, F., Predicting core level binding energies shifts: Suitability of the projector augmented wave approach as implemented in VASP. *J. Comput. Chem.* **2017,** *38*, 518-522.

22. Göransson, C.; Olovsson, W.; Abrikosov, I. A., Numerical investigation of the validity of the Slater-Janak transition-state model in metallic systems. *Phys. Rev. B* **2005,** *72*, 134203.

23. Köhler, L.; Kresse, G., Density functional study of CO on Rh(111). *Phys. Rev. B* **2004,** *70*, 165405.

24. Suh, I.-K.; Ohta, H.; Waseda, Y., High-temperature thermal expansion of six metallic elements measured by dilatation method and X-ray diffraction. *J. Mater. Sci.* **1988,** *23*, 757-760.

25. Hsieh, T.-E.; Frisch, J.; Wilks, R. G.; Bär, M., Unravelling the Surface Oxidation-Induced Evolution of the Electronic Structure of Gallium. *Appl. Mater. Interfaces* **2023,** *15*, 47725–47732.

26. Sahoo, S. R.; Ke, S.-C. Spin-Orbit Coupling Effects in Au 4f Core-Level Electronic Structures in Supported Low-Dimensional Gold Nanoparticles *Nanomaterials* [Online], 2021.

27. Baren, M. R., The Ag-Ga (Silver-Gallium) system. *Bull. Alloy Phase Diagr.* **1990,** *11*, 334-339.

28. Dürrwächter, M.; Indlekofer, G.; Boyen, H. G.; Oelhafen, P.; Quitmann, D., Core level binding energy shifts in liquid binary alloys: Au-Ga. *J. Non-Cryst. Solids* **1993,** *156-158*, 241-245.

29. Wittkämper, H.; Maisel, S.; Moritz, M.; Grabau, M.; Görling, A.; Steinrück, H. P.; Papp, C., Surface Oxidation-induced Restructuring of Liquid Pd-Ga SCALMS Model Catalysts. *Phys. Chem. Chem. Phys.* **2021,** *23*, 16324-16333.

30. Wittkämper, H.; Maisel, S.; Wu, M.; Frisch, J.; Wilks, R. G.; Grabau, M.; Spiecker, E.; Bär, M.; Görling, A.; Steinrück, H.-P.; Papp, C., Oxidation Induced Restructuring of Rh–Ga SCALMS Model Catalyst Systems. *J. Chem. Phys.* **2020,** *153*, 104702.
